# Comparative morphological analysis of the zonular apparatus in porcine, feline, and canine species

**DOI:** 10.3389/fmed.2025.1717670

**Published:** 2026-01-14

**Authors:** Ye Lu, Kuankuan Wu, Zhiqiao Liang, Kun Lv, Zeyuan Wang, Fengrui Yang, Yitong Hu, Huijuan Wu

**Affiliations:** 1 Department of Ophthalmology, Peking University People’s Hospital, Beijing, China; 2 Beijing Key Laboratory of Diagnosis and Therapy of Retinal and Choroid Diseases, College of Optometry, Eye Diseases and Optometry Institute, Peking University Health Science Center, Beijing, China

**Keywords:** across species, animal model, glaucoma, vitreous zonules, zonular apparatus

## Abstract

**Purpose:**

To characterize and compare the morphology of the zonular apparatus in pigs, cats, and dogs to identify the optimal animal model for studying zonular pathophysiology in angle-closure mechanisms.

**Methods:**

Porcine (*n* = 15), feline (*n* = 8), and canine (*n* = 4) eyes were included in this descriptive, exploratory study. Slit-lamp biomicroscopy, photography, and histological staining (Hematoxylin and Eosin, Masson’s Trichrome) were utilized to evaluate structure and collagen distribution. The anterior segments of porcine, feline, and canine eyes were imaged *ex vivo* using ultrasound biomicroscopy (UBM). Ultrastructural morphology was further analyzed with scanning electron microscopy (SEM). Quantitative measurements were obtained with ImageJ and CaseViewer v2.4. This study is descriptive and that no inferential statistics were performed.

**Results:**

Significant interspecies differences were observed in zonular fiber density, orientation, and insertion patterns. The sagittal width of posterior vitreous zonule region was notably narrower than in humans (3–4 mm): approximately 0.56 mm in pigs, 0.40 mm in cats, and 0.30 mm in dogs. UBM imaging successfully detected vitreous zonules in porcine and feline eyes, but failed to visualize them in canine eyes. Dogs and cats exhibited similar zonular and ciliary body morphology; however, both species displayed leaf-shaped ciliary bodies and looser zonular arrangements compared to the more compact porcine configuration. Ultrastructural analysis using SEM identified consistent vitreous zonule architecture in all species.

**Conclusion:**

Among the three species examined, unique zonular fiber origins and the ubiquitous vitreous zonules support the use of pigs or cats in studies of accommodation and glaucoma pathogenesis.

## 1 Introduction

**FIGURE 1:**
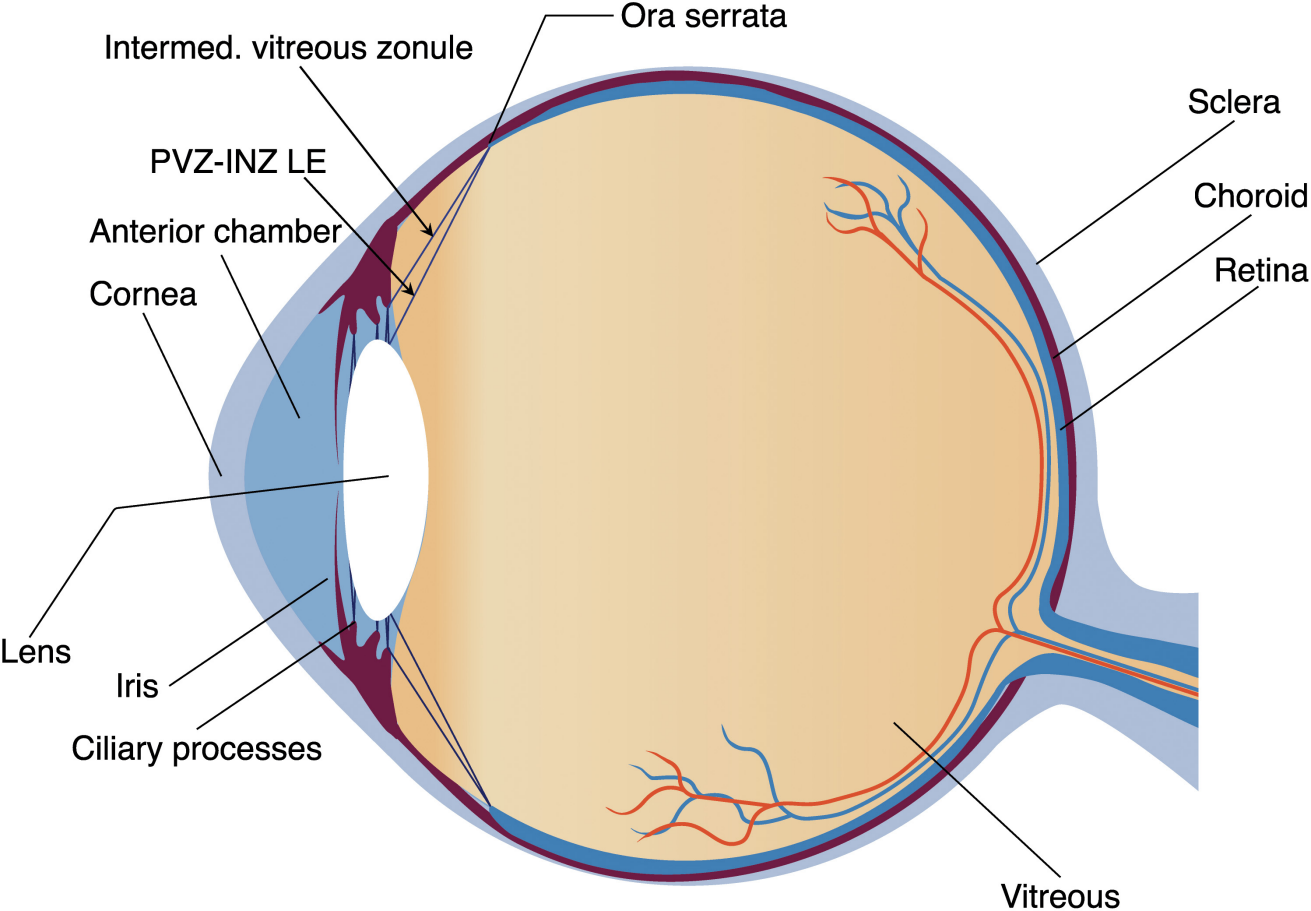
Diagram of mammalian eyeball. The arrows indicate the fiber bundles extending from the posterior ciliary plicata to the vitreous membrane near the ora serrata. These bundles change direction and attach directly to the posterior lens equator, designated as the posterior vitreous zonules insertion zone and posterior lens equator (PVZ INS-LE). Both regions are classified as vitreous zonules.

The vitreous zonule (VZ) is morphologically characterized by interconnecting fiber networks that extend from the zonular plexus within the interciliary valleys of the posterior pars plicata to the vitreous base near the ora serrate (Figure 1)(1–3). Ultrastructural analysis using scanning electron microscopy (SEM) has revealed a tripartite organization of the VZ in primates, consisting of anterior, intermediate, and posterior fiber groups. Importantly, these distinct structures can also be visualized *in vivo* with ultrasound biomicroscopy (UBM) (4). Comprehensive investigations of human VZ morphology using UBM, anterior segment optical coherence tomography, and SEM, have established key anatomical features that serve as morphological benchmarks for evaluating structural homology in animal models (1, 3–5).

The VZ has been implicated in the pathogenesis of different forms of glaucoma. Initially, Kaufman et al. proposed that VZ-mediated aqueous humor dynamics during ciliary muscle contraction might contribute to the development of primary open-angle glaucoma (6). More recent evidence, however, has highlighted the VZ’s significance in angle-closure pathogenesis. Shon et al. examined the relationship between the VZ and anterior chamber angle configuration in patients with primary angle-closure (PAC) and PAC glaucoma (PACG), discovering that eyes lacking a detectable VZ had significantly narrower angles (5). Subsequently, Lv et al. demonstrated a decreased prevalence of visible VZ in individuals with PAC and PACG compared to age- and sex-matched controls, further substantiating the link between VZ and primary angle-closure disease (PACD) (4).

Animal models are indispensable in ophthalmic research, particularly for elucidating the pathogenesis of glaucoma and exploring novel therapeutic strategies (7). While non-human primates are considered the gold standard due to their close anatomical and metabolic resemblance to humans, their use is limited by significant ethical and logistical constraints. Alternatively, murine models offer advantages in cost-effectiveness, ease of husbandry, and amenability to genetic modification, enabling precise experimental manipulation. However, their small ocular size poses technical challenges for microsurgical procedures and *in vivo* imaging applications (8). Furthermore, studies investigating the relationship between VZ and PACD remain insufficient. Therefore, identifying animal models with intact VZ systems that closely mirror human anatomy is essential for studies of the VZ’s role in PACD.

The aims of this study are to characterize interspecies (porcine, feline, and canine) differences in zonular apparatus structure and to identify animal models exhibiting the highest degree of structural homology with humans, thereby establishing optimal models for investigating novel pathogenesis in glaucoma.

## 2 Materials and methods

### 2.1 Eyeballs preparation

Fifteen fresh, normal eyes from pigs of either sex, aged 6–8 months, were obtained from a local slaughterhouse (Shunxin Agricultural Co., Ltd., Pengcheng Food Branch, Beijing, China) and immediately transported to the laboratory on ice. All porcine specimens were processed within 2 h of enucleation to preserve tissue quality for subsequent analyses.

The feline group consisted of two 1-year-old females, one 1-year-old male, and one 8-year-old male. The canine group included three females and one male, with ages of 2, 4, 5 and 14 years. Then, 12 eyes were collected from cats (*n* = 8) and dogs (*n* = 4) that had been euthanized humanely via intravenous injection of sodium pentobarbital for non-ocular clinical conditions unrelated to this study.

In subsequent experiments, six *ex vivo* porcine eyes, two *ex vivo* feline eyes, and one *ex vivo* canine eye were allocated for UBM imaging. Seven porcine eyes, four feline eyes, and one canine eye were prepared for scanning electron microscopy. Additionally, one eye from each species was processed for hematoxylin-eosin and Masson’s trichrome staining, and another set consisting of one porcine, one feline, and one canine eye was used for slit-lamp microscopy photography.

Post-mortem examination confirmed that all animal eyes were anatomically normal without structural abnormalities. The use of animal specimens was approved by the Ethics Review Committee of Peking University People’s Hospital (Approval Number: 2025PHE065). All procedures adhered to the ARVO Statement for the Use of Animals in Ophthalmic and Vision Research and complied with institutionally approved animal protocols.

### 2.2 Ultrasound biomicroscopy imaging

The anterior segments of porcine, feline, and canine eyes were imaged *ex vivo* using UBM. UBM scanning (Aviso; Quantel Medical, Inc., Bozeman, MT, United States) was performed using a 50-MHz transducer by a trained operator (Y.L) to visualize the VZ and ciliary body. The UBM images obtained in this study were independently evaluated by two experienced observers (Y.L. and KK.W) to determine the presence of the vitreous ligament. Inter-observer agreement analysis yielded a kappa value of 0.78 (*P* = 0.016), indicating substantial consistency between the evaluators. Each globe was positioned in a custom-designed chamber filled with sterile water, with the fluid level maintained approximately 1 cm above the apex of the eyeball. Images were acquired from multiple quadrants to visualize the VZ. During dynamic real-time scanning, only images with clearly visible VZ structures were captured and stored for analysis.

### 2.3 Morphology analysis

#### 2.3.1 Gross anatomy

Following equatorial transection and removal of the posterior segment, the anterior ocular segment was dissected into wedge-shaped sections encompassing the ciliary body, limbal corneoscleral junction, and zonule-lens-vitreous interface. These sections were examined under a stereomicroscope, and gross anatomical images were acquired using a digital camera (Canon EOS 1500D, Canon Inc., Tokyo, Japan).

#### 2.3.2 Scanning electron microscope

Following gross anatomical examination, tissues were sectioned into quadrants using established protocols optimized for zonular fiber visualization (1). Specimens were then fixed in 2.5% (v/v) glutaraldehyde (Beijing Biotoppted Science & Technology Co., Ltd., Cat# Top0639) in PBS (0.01 M, pH 7.4) at 4°C for 24 h, post-fixed in 1% osmium tetroxide, dehydrated through a graded acetone series, critical-point dried (HITACHI ES-2030), sputter-coated with gold-palladium (Au/Pd), and examined by SEM (FEI, INSPECT S50) at various magnifications.

#### 2.3.3 H&E and Masson staining

Fresh enucleated eyes were fixed in ferrous ammonium sulfate solution (Servicebio, China, Cat#G1109) at room temperature for 24 h, then processed for routine paraffin embedding. Serial sections (3 μm) were stained with hematoxylin and eosin (Servicebio, China; Cat. G1003) or Masson’s trichrome (Servicebio, China; Cat. G1006) following standard protocols. Histological images were captured using an upright optical microscope (Nikon, Japan).

### 2.4 Image quantification and statistical analysis

Morphometric assessment of zonular fibers (length, width, and spacing) was performed on SEM and histological images using ImageJ v1.54g (NIH, United States) and CaseViewer v2.4 (3DHISTECH Ltd., Hungary). The measurements of the interval of intermediate vitreous zonules of porcine and feline are presented as mean ± standard deviation. Statistical comparisons between groups were performed with an independent samples *t*-test. A 95% confidence interval was applied, and a *p* < 0.05 was deemed statistically significant. Additionally, observer agreement was assessed and reported as a kappa value. Other data are reported as approximate ranges to account for interspecies anatomical variability.

## 3 Results

### 3.1 Comparative histomorphology of zonular fibers and angle structures

**FIGURE 2:**
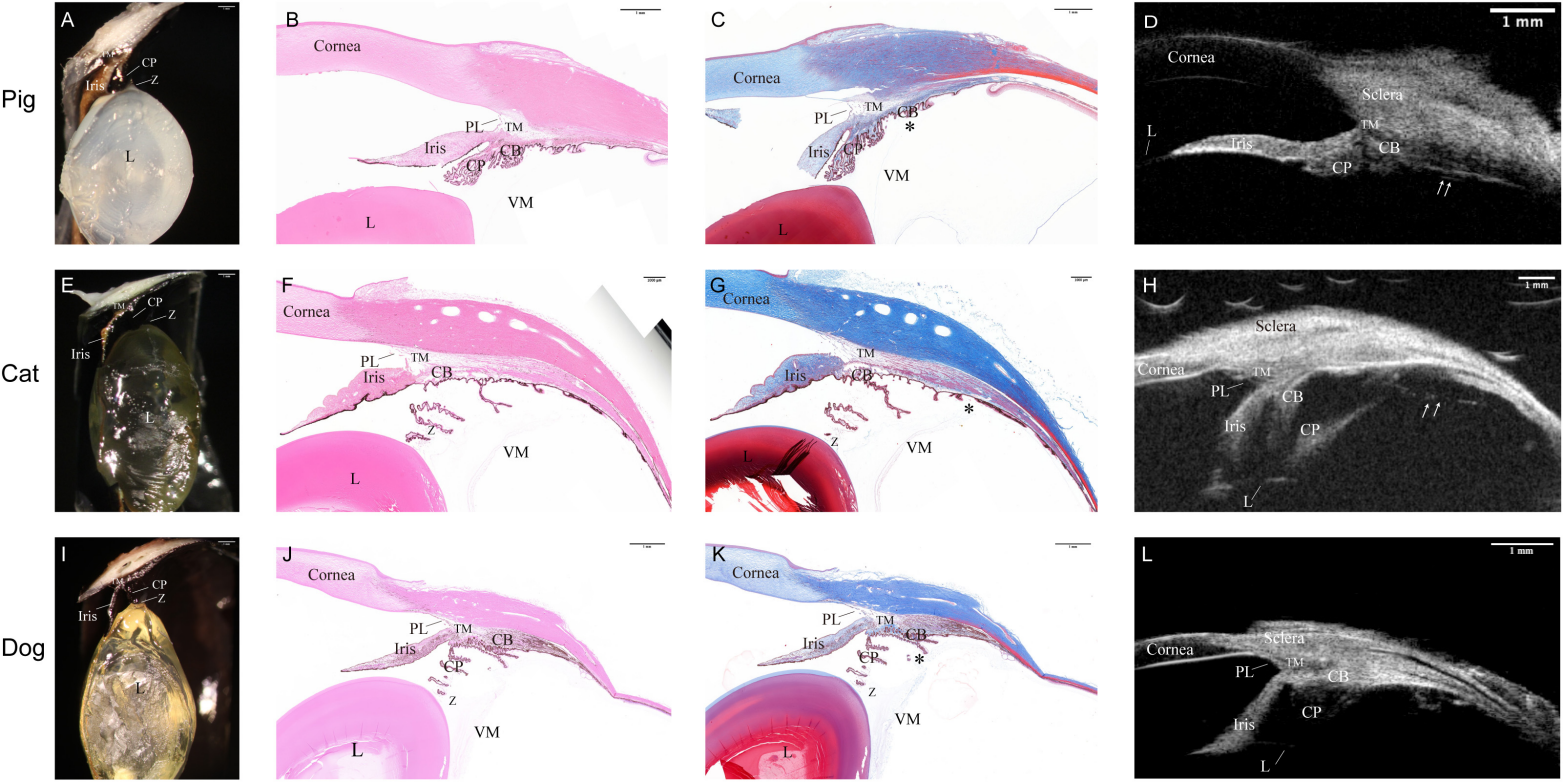
Morphology of the zonular apparatus across three species. Microscopic images reveal similar configurations of the zonular apparatus in porcine **(A)**, feline **(E)**, and canine **(I)** eyes. Insets **(B,F,J)** show sagittal sections of the zonular apparatus stained with Hematoxylin and Eosin. Insets **(C,G,K)** present sagittal sections stained with Masson’s trichrome. The sagittal width of posterior vitreous zonule region measures approximately half the distance to the termination of Descemet’s membrane in pigs (star symbol areas in **C,G,K**). Specifically, the width of this attachment zone is 0.56 mm in pigs, 0.40 mm in cats, and 0.30 mm in dogs. *Ex vivo* UBM results revealed a discrete vitreous zonule in porcine and feline eyes (white arrow in **D,H**), whereas canine eyes displayed short, bristle-like projections on the pars plana surface **(L)**. CB, ciliary body; CP, ciliary processes; L, lens; PL, pectinate ligament; TM, trabecular meshwork; VM, vitreous membrane; Z, zonule.

The histological characteristics of the suspensory apparatus across all examined species are depicted in Figure 2. Notably, both the zonule and VM exhibited comparable gross morphology and anatomical relationships among the three species. Sagittal dissection and *ex vivo* UBM images revealed that porcine CPs adopt an ellipsoidal cylindrical configuration (Figures 2B–D), akin to that of humans. In contrast, feline (Figures 2F–H) and canine CPs (Figures 2J–L) assumed a distinct leaf-shaped form. The sagittal width of posterior vitreous zonule region was roughly half the distance from Descemet’s membrane termination to the ora serrata. This distance measured around 4.5 mm in pigs, 7.4 mm in cats, and 6.6 mm in dogs, as indicated by the star symbol areas in Figures 2C,G,K. However, this attachment zone was considerably narrower than in humans measuring about 0.56 mm in pigs, 0.40 mm in cats, and 0.30 mm in dogs, compared to 3∼4 mm in humans. Furthermore, *ex vivo* UBM scaning revealed a discrete VZ in porcine and feline specimens (indicated by white arrows in Figures 2D,H), whereas canine eyes displayed short, bristle-like projections on the pars plana surface (Figure 2L).

### 3.2 Porcine zonule system

**FIGURE 3:**
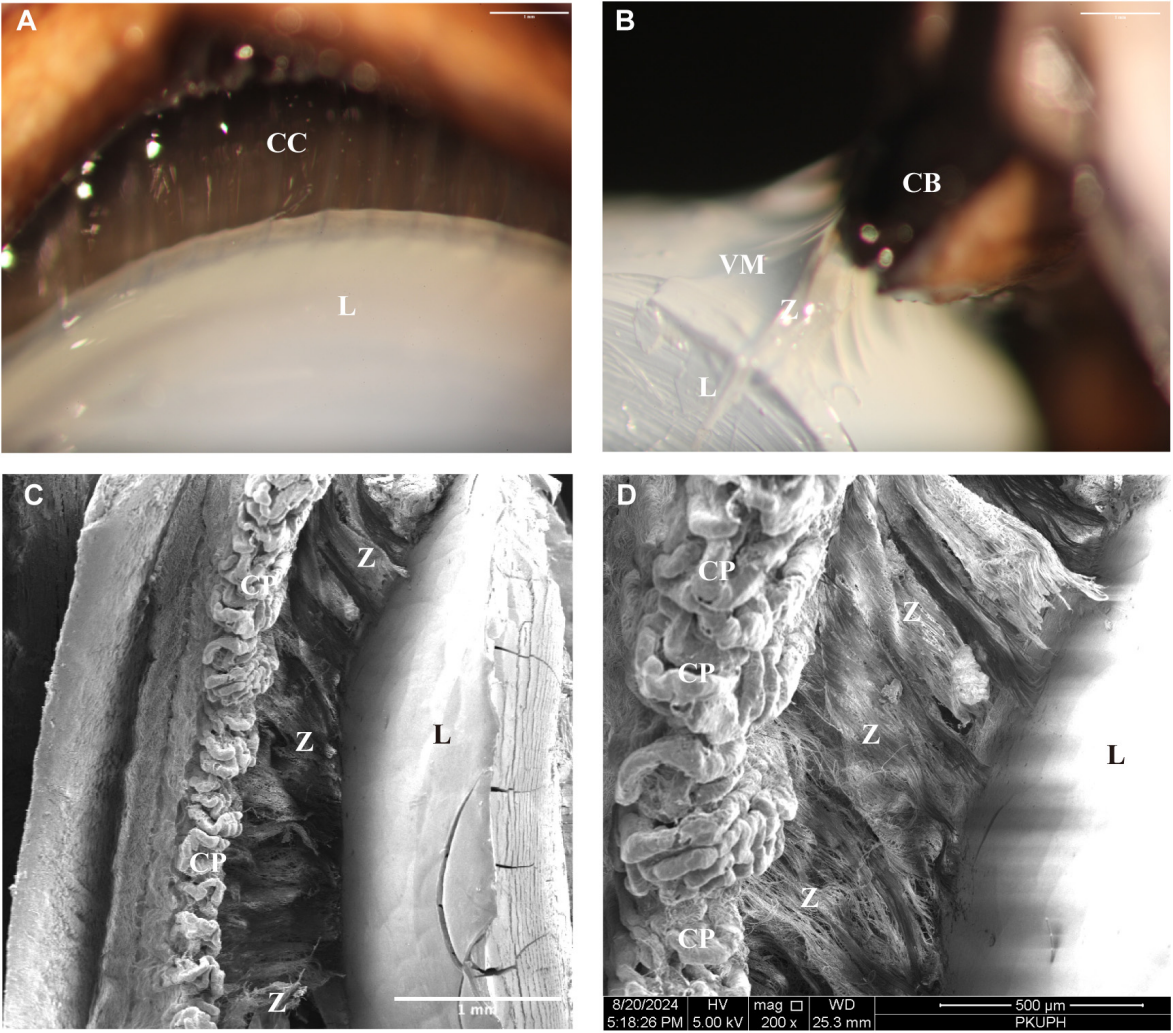
**(A)** Anterior view of the porcine lens of showing zonular filaments originate from the ciliary body and insert into the lens equator. **(B)** Sagittal lens profile demonstrating densely packed fibers from the valleys and ridges of the pars plicata forming the translucent, lamellar structures. **(C,D)** Scanning electron micrographs at varying magnifications showing fibers from the pars plicata form laminae that insert parallel to the lens equator. CB, ciliary body; CC, ciliary crown; CP, ciliary processes; L, lens; VM, vitreous membrane; Z, zonule.

**FIGURE 4:**
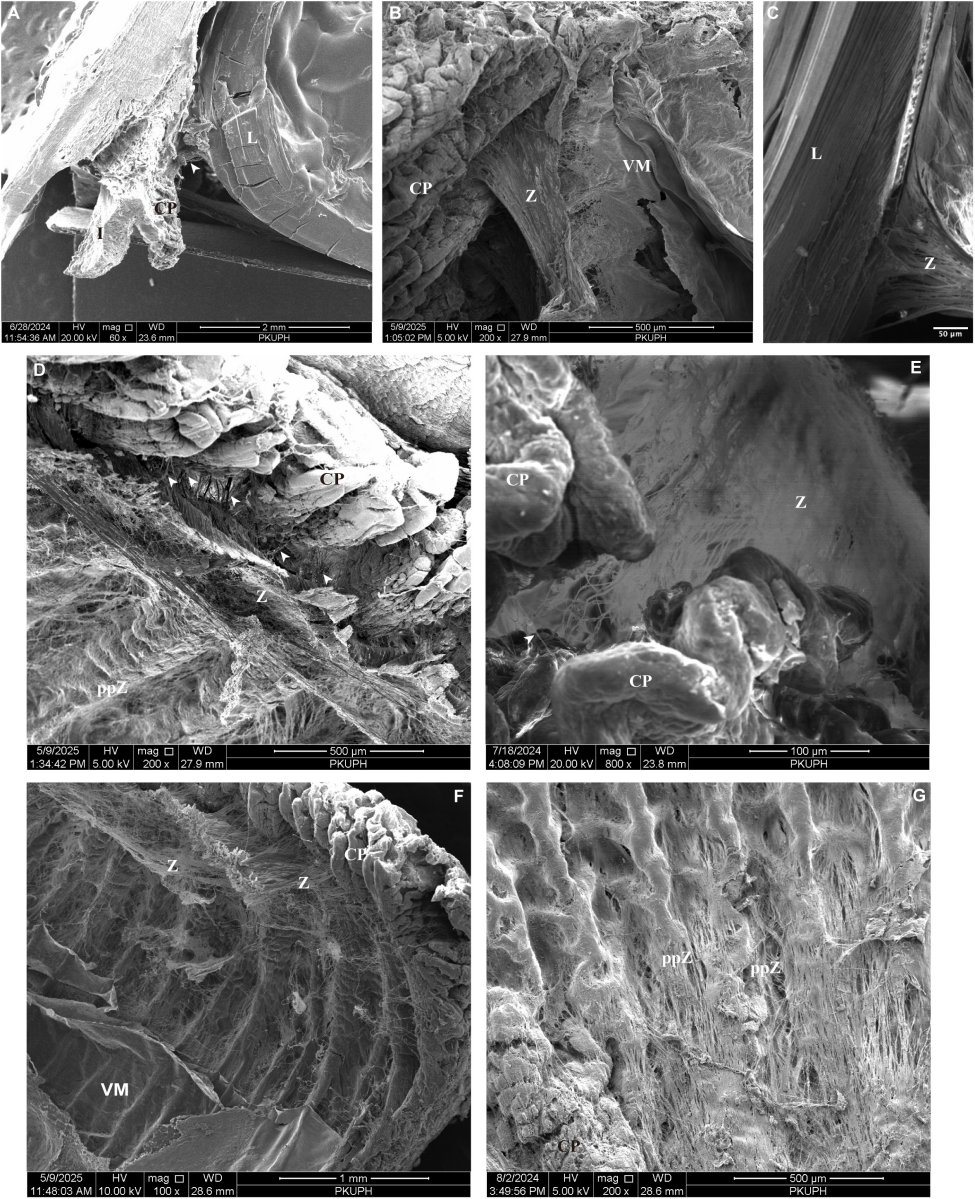
**(A)** Scanning electron micrograph of the sagittal section through the porcine zonular apparatus. White arrow indicates the lens zonular fibers. **(B)** Porcine anterior zonular fibers originate from valleys of the pars plicata, with fibers from valleys converge into wedge-shaped sheets. **(C)** Porcine anterior zonular fibers insert perpendicularly into the lens equator in a wedge-like manner. **(D)** From an anterior lens view, zonular filaments originate from valleys and ridges of the pars plicata, approximately 0.35–0.4 mm posterior to the base of ciliary processes. **(E)** Interconnecting fibers become increasingly distinct at higher magnification, with sparse interconnections between valleys of the pars plicata. **(F)** Zonular fibers extend anteriorly from pars plana toward the lens. **(G)** Within the pars plana, zonular fibers are predominantly oriented longitudinally and regularly interlace to form an interwoven mat-like network, anchored to the ciliary epithelium via the internal limiting membrane. CP, ciliary processes; L, lens; ppZ, pars plana zonule; VM, vitreous membrane; Z, zonule.

From an anterior lens perspective, all zonular filaments arise from the ciliary body approximately 0.35–0.4 mm posterior to the base of CPs and insert into the lens equator (Figures 3, 4A–D). In the porcine eye, anterior zonular fibers originate from both the valleys and ridges of the pars plicata. Fibers derived from the valleys converge to form wedge-shaped sheets that insert perpendicularly into the lens equator, spanning approximately 0.28 mm (Figure 4C). This characteristic sheet-like morphology (Figure 4B) corresponds to the distinct outlines observed in gross microscopic anatomy (Figure 3B). Conversely, fibers from the ridges of the pars plicata also generate laminae that insert into the lens equator parallel to its plane (Figures 3C,D, 4D). Densely packed fibers originating from both the valleys and ridges of the pars plicata give rise to the translucent, lamellar structures seen in gross microscopy (Figure 3A). Throughout its length, the zonular network maintains generally organized in a parallel manner (Figure 3D), with interconnecting fibers becoming increasingly prominent at higher magnification (Figure 4E). Notably, sparse interconnections are evident between the valleys of the pars plicata (Figure 4E).

Within the pars plana of the ciliary body, zonular fibers are predominantly oriented longitudinally and regularly interlace to form an interwoven, mat-like network anchored to the ciliary epithelium via the internal limiting membrane (Figure 4G). As demonstrated in Figures 4D,F, these zonular fibers extend anteriorly from pars plana toward the lens.

**FIGURE 5:**
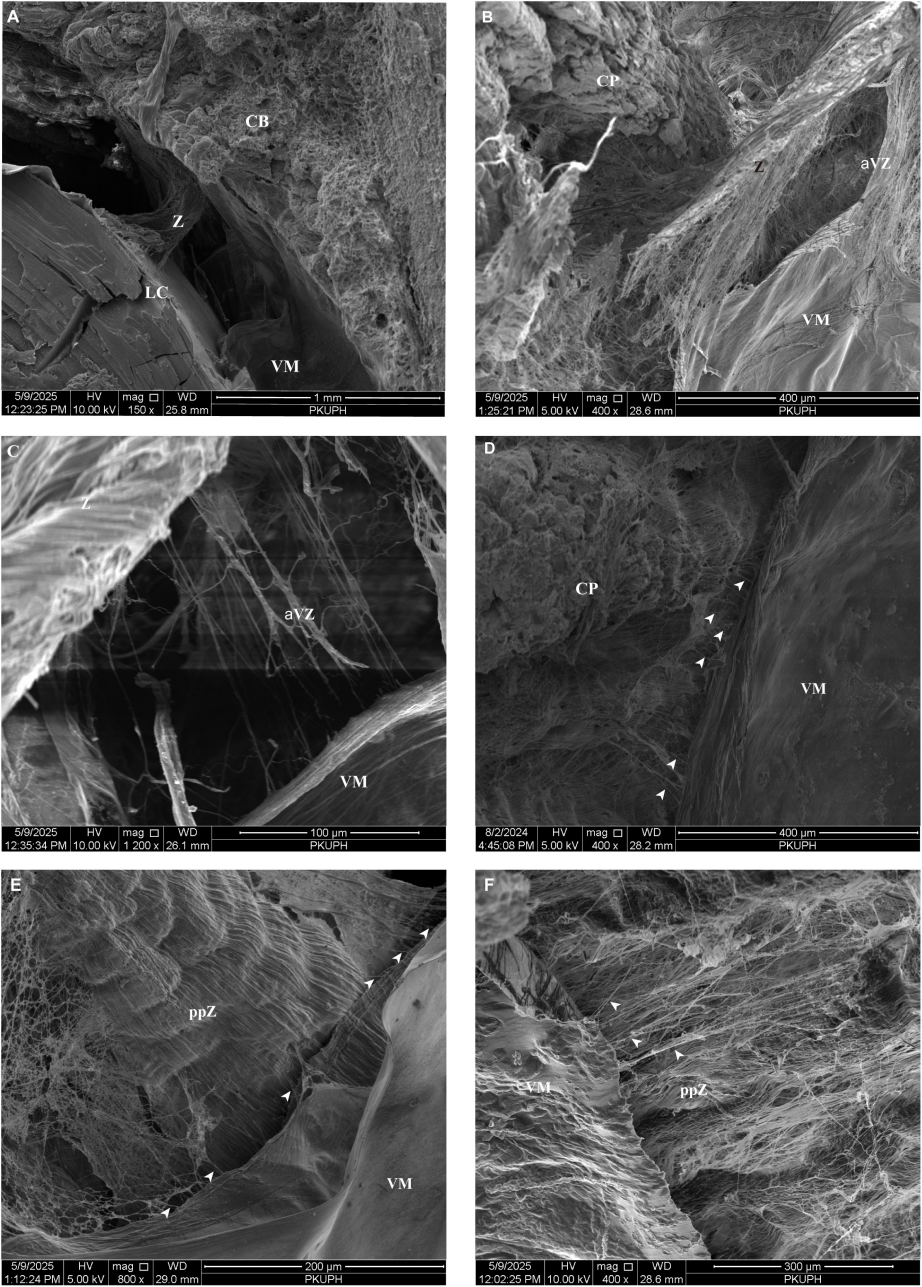
**(A)** Scanning electron micrograph of a sagittal section through the porcine zonular apparatus. **(B)** Anterior-view scanning electron micrograph of the zonular apparatus showing the vitreous membrane and zonular lamella converging at a shared insertion site into the lens capsule. **(C)** Zonular fibers from the zonular plexus insert into the vitreous membrane at intervals of approximately 2–25 μm, forming discrete linkages defined as anterior vitreous zonules. **(D)** Lifting the vitreous membrane over the posterior pars plicata and mid-pars plana revealed a cleft between the vitreous membrane and the pars plana zonules, bridged by fiber bundles known as intermediate vitreous zonules (white arrows). These bundles are irregularly spaced around the posterior pars plicata and the circumference of the pars plana at intervals of approximately 4–20 μm. **(E)** After elevating the vitreous membrane over the mid-pars plana, the intermediate vitreous zonules (white arrows) were found to be uniformly spaced around the posterior pars plicata and entire pars plana circumference. **(F)** At the ora serrata, each intermediate vitreous zonules bundle divides into fine fibrils that merge into the vitreous membrane. Together with their posterior extensions, the vitreous membrane and intermediate vitreous zonules attach to the pars plana zonule via numerous obliquely oriented fine fibrils. This forms a multilayered, interconnected complex at the posterior attachment site of the vitreous to the pars plana and ora serrata, designated as the posterior vitreous zonule (white arrows). aVZ, anterior vitreous zonules; CP, ciliary processes; L, lens; LC, lens capsule; ppZ, pars plana zonule; VM, vitreous membrane; Z, zonule.

Microscopic examination of the attachment sites of the VM and zonular lamina revealed that both structures insert into the posterior lens capsule at the lens periphery (Figures 5A,B). When the VM was elevated from the zonular lamina, their insertion areas appeared closely associated; however, the VZ consistently overlies the zonular lamina. Similar to the zonular fibers, VZ fibers interdigitate to form a continuous, sheet-like structure.

Zonular fibers originating from the zonular plexus insert periodically into the VM at intervals of approximately 4–25 μm with an average value of 13.06 μm (Figure 5C), forming discrete linkages defined as anterior vitreous zonules (aVZ) (1). Careful lifting of the VM over the posterior pars plicata and the mid-pars plana (without complete removal) revealed a distinct cleft between the VM and pars plana zonules. This cleft is spanned by fiber bundles referred to as intermediate vitreous zonules (iVZ) (1). These iVZ bundles are irregularly spaced around the posterior pars plicata and pars plana at intervals of 17.62 ± 1.77μm (*n* = 3) (Figures 5D,E). At the ora serrata, each iVZ bundle divides into fine fibrils that merge into the VM (Figure 5F).

The VM and iVZ, together with their posterior extensions, attach to the pars plana zonule via numerous obliquely oriented fine fibrils (Figures 5E,F). At the posterior attachment site where the vitreous connects to the pars plana and ora serrata regions, these structures collectively form a multilayered, interconnected complex designated as the posterior vitreous zonule (pVZ) (1). The porosity of pVZ was measured at 20.75%.

### 3.3 Feline zonule system

**FIGURE 6:**
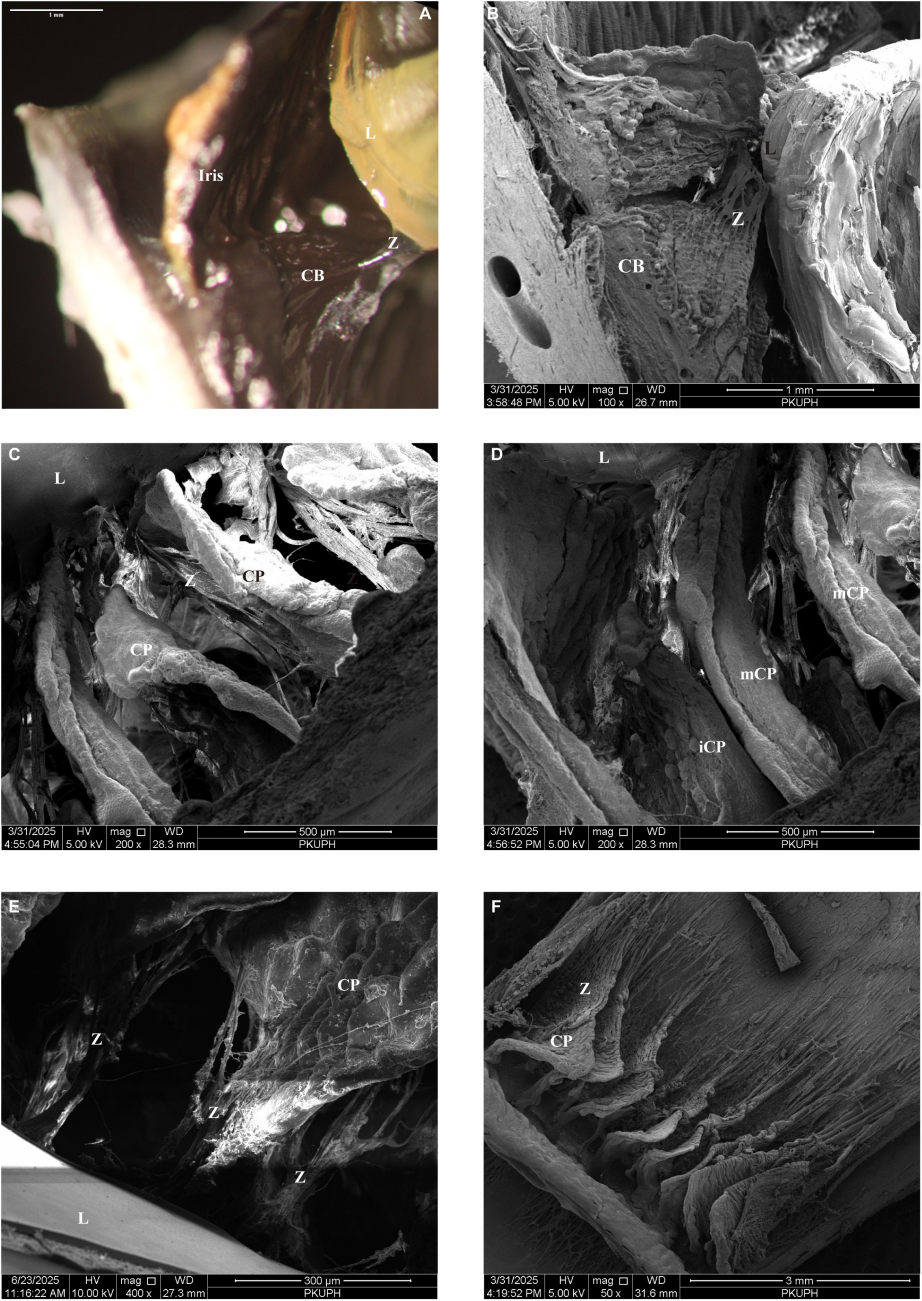
**(A)** The gross anatomic relationship between the lens and ciliary body of a feline eye. **(B)** Scanning electron microscopy of the lens and ciliary body in a feline sagittal section. **(C)** The ciliary processes can be classified into two types: major ciliary processes and intermediate ciliary processes. Each major ciliary process generates two discrete fiber bundles: one originating from the crest and another extending from the lateral walls. **(D)** The major ciliary processes display a uniform-thickness, leaf-like morphology. Principal zonular fibers in felines are more sparsely distributed, with spacing that matches the intervals between CPs and/or plicae **(E)** Zonular fibers establish insertion sites approximately 0.3–0.7 mm posterior to the anterior border of the pars plicata. Furthermore, these fibers converge to create wedge-shaped lamellae that extend approximately 0.16 mm and insert perpendicularly into the equatorial region of the lens. **(F)** Following removal of the lens and vitreous membrane, the density of zonular mesh progressively decreases, thereby exposing underlying epithelial architecture. CP, ciliary processes; L, lens; LC, lens capsule; mCP, major ciliary process; iCP, intermediate ciliary process; VM, vitreous membrane; Z, zonule.

The gross anatomic relationship between the lens and ciliary body is illustrated in Figures 6A,B. As demonstrated in Figures 6C,D, the CPs can be classified into two distinct types: major CPs and intermediate CPs. Major CPs (Figure 6D) display uniform thickness and leaf-like morphology. In contrast, the smaller intermediate CPs, which correspond to typical ciliary plicae, are usually found singly in the central region between adjacent major CPs.

Each major CP generates two discrete fiber bundles: one originating from the crest and another extending from the lateral walls (Figures 6C,D). These fibers groups remain anatomically separate from each other. Additionally, zonular fibers are present within intermediate CPs and within the solitary plicae located between major CPs. In contrast to the densely organized zonular laminae observed in porcine eyes, principal zonular fibers in felines are more sparsely distributed, with spacing that matches the intervals between CPs and/or plicae (Figures 6C,D). These fibers establish insertion sites approximately 0.3–0.7 mm posterior to the anterior border of the pars plicata. Furthermore, these fibers converge to create wedge-shaped lamellae that extend approximately 0.16 mm and insert perpendicularly into the equatorial region of the lens (Figure 6E).

**FIGURE 7:**
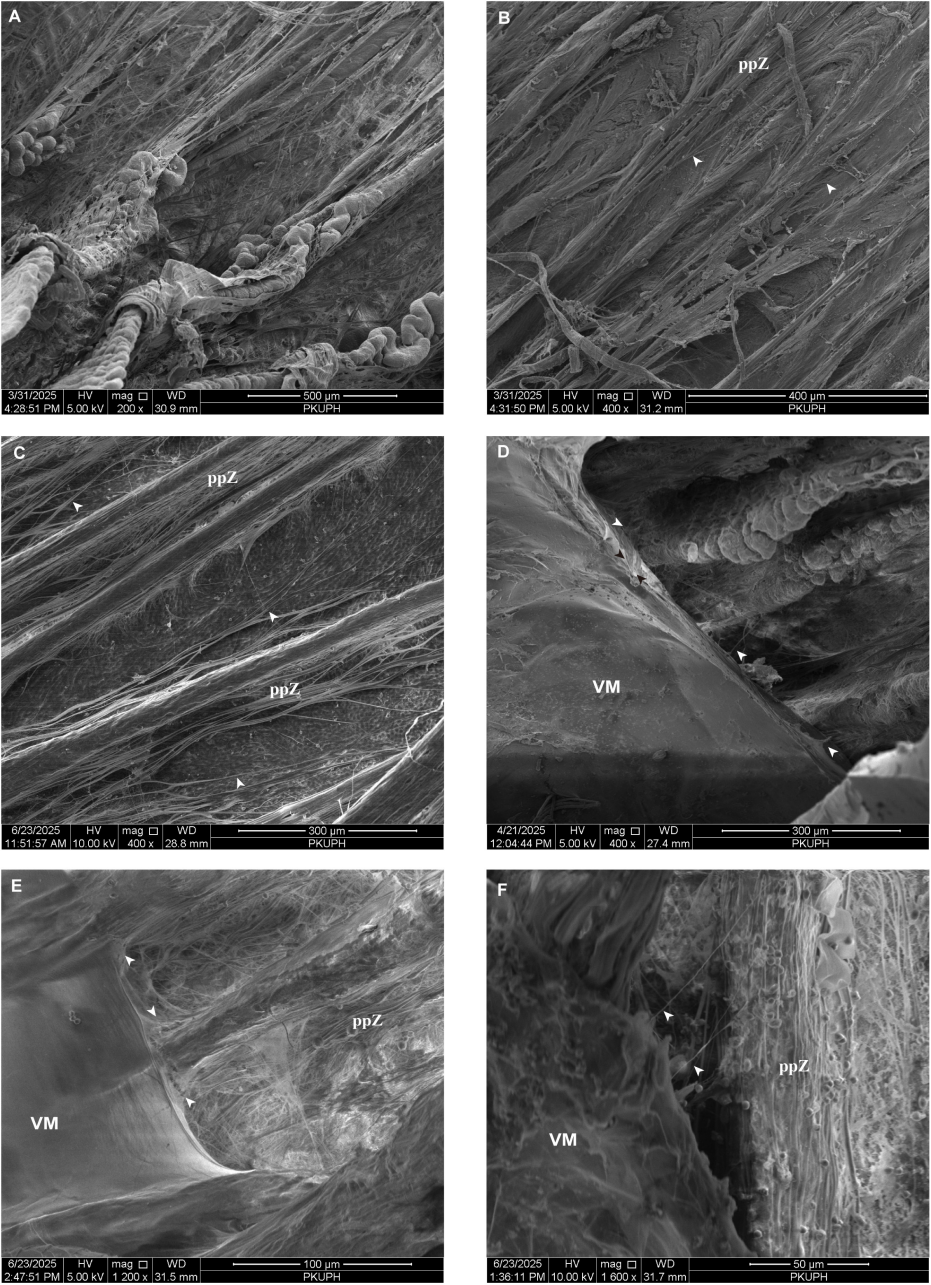
**(A)** Fiber bundles bifurcate in accordance with local anatomical features: some follow the ramifications of ciliary process, while others course along divergent posterior plicae. The majority of fibers maintain parallel alignment with major processes/plicae. **(B)** The larger superficial fibers extend parallel across the pars plana, typically terminating near the ora ciliaris retinae. **(C)** Certain fibers adopt oblique trajectories, traversing adjacent plicae (white arrows). Notably, the frequency of branching and anastomosis is significantly reduced among the larger fibers in this region. **(D)** Distinct fibrous connections between the vitreous membrane and the ciliary processes (white arrows). These fibers originate from the vitreous membrane as intermediate vitreous zonules fibers (black arrows). The points of insertion for the intermediate vitreous zonules fibers into the vitreous membrane are spaced at intervals ranging from 2.5 to 31.3 μm. **(E)** Intermediate vitreous zonules converge upon the ciliary processes to form a wedge-shaped reticular structure (white arrows). **(F)** At specific sites, the vitreous membrane establishes connections to the valleys between adjacent ciliary processes (white arrows). ppZ, pars plana zonule; VM, vitreous membrane.

As these fibers extend posteriorly along feline CPs—unlike the pattern observed in porcine eyes—the density of the zonular mesh progressively decreases, exposing underlying epithelial structures (Figures 6F, 7A). Fiber bundles bifurcate according to local anatomical features: some follow the ramifications of CPs, while others course along divergent posterior plicae. Although most fibers maintain a parallel alignment with major processes and plicae (Figures 7A,B), certain fibers adopt oblique trajectories, traversing adjacent plicae (Figure 7C). Importantly, the frequency of branching and anastomosis is significantly reduced among the larger fibers in this region (Figure 7C). The larger superficial fibers extend to the pars plana and typically terminate near the ora ciliaris retinae (Figure 7B).

The spacing of aVZ ranged from 15 to 38 μm, with an average value of approximately 27.25 μm. Careful elevation of the VM in the feline eye, exposing the region adjacent to the CPs near the pars plana, reveals distinct fibrous connections between the VM and the CPs (white arrows in Figure 7D). These fibers originate from the VM as iVZ fibers (black arrow in Figure 7D). These iVZ bundles are irregularly spaced around the posterior pars plicata and pars plana at intervals of 16.26 ± 2.16μm (*n* = 3). The spacing of the iVZ was numerically greater in pigs than in cats, and the iVZ appeared more densely arranged in the feline samples. However, this difference was not statistically significant (*t*-test, *P* = 0.67). Additionally, at specific sites, the VM establishes connections to the valleys between adjacent CPs (Figure 7F). The points of insertion for the iVZ fibers into the VM are spaced at intervals ranging from 2.5 to 31.3 μm. The porosity of pVZ was measured at 30.90% (Figure 7E).

### 3.4 Canine zonule system

**FIGURE 8:**
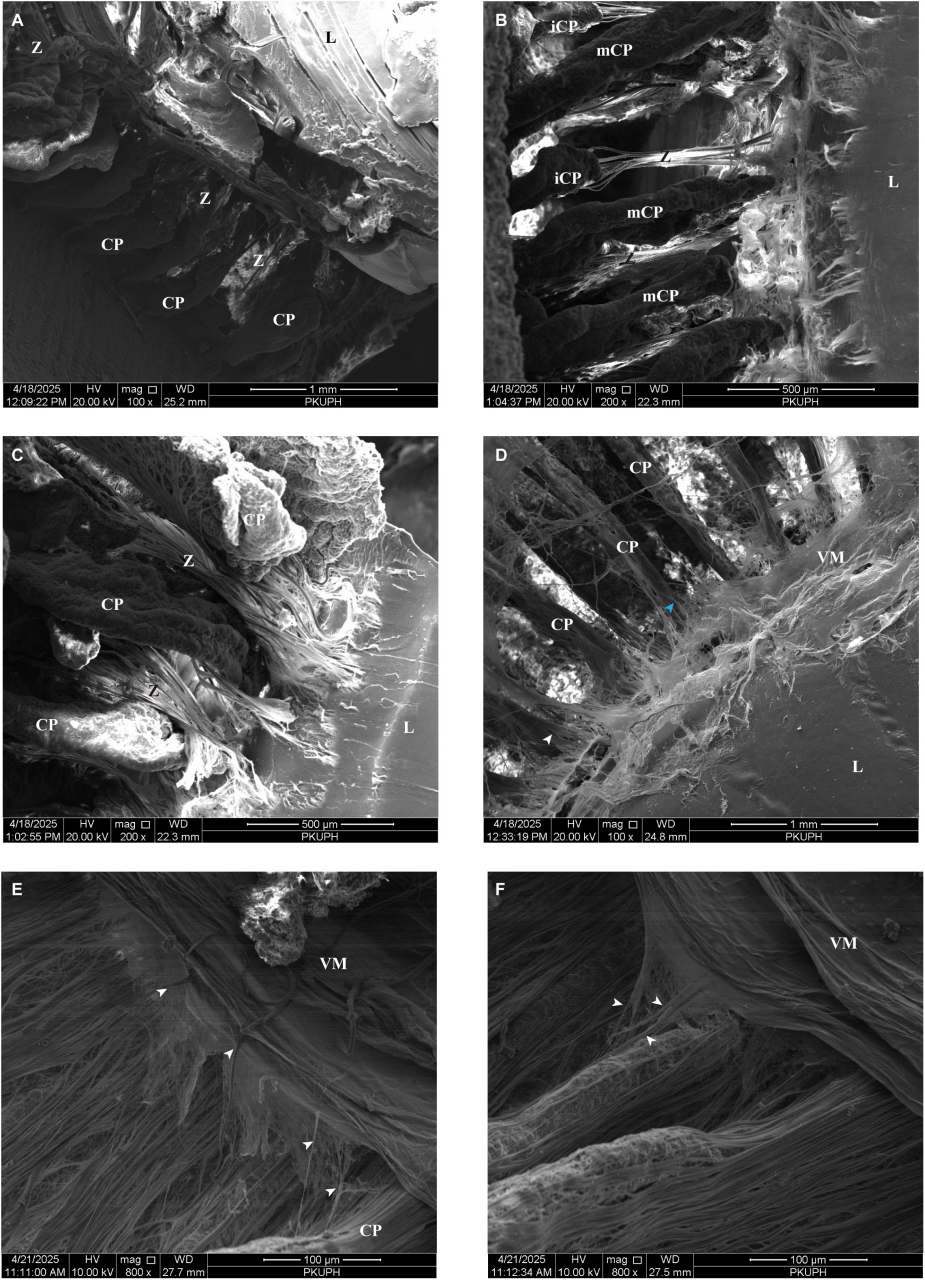
**(A)** Scanning electron microscopy reveals the anatomical features of the lens and ciliary body in a canine sagittal section. An anterior view of the lens demonstrates that zonular fibers consolidate into larger bundles at distances of approximately 0.26–0.5 mm posterior to the anterior edge of the ciliary processes before inserting into the lens capsule **(B)** Similar to the feline configuration, canine eyes possess both major and intermediate ciliary processes. **(C)** Zonule fibers originate from both the crests of ciliary processes and the valleys between them, subsequently projecting toward the lens capsule. **(D)** When viewed from the posterior aspect of the lens, the anterior vitreous zonules (blue arrow) are situated in a distinct anatomical plane, separate from the primary lens zonular apparatus (white arrow). This spatial arrangement closely parallels that observed in porcine eyes, where the anterior vitreous zonule insert into the lens capsule at similarly positioned sites near the lens equator. **(E)** Further dissection of the vitreous membrane, extending to the posterior portion of the ciliary processes, reveals the presence of intermediate vitreous zonules (white arrows). The canine intermediate vitreous zonule establish fibrous connections with the pars plana zonules. These interconnections exhibit reciprocal weaving, resulting in an intricate reticular network. **(F)** This reticular fiber arrangement (white arrows) overlies both the crestal ridges and inter-process valleys of canine ciliary processes. CP, ciliary process; L, lens; mCP, major ciliary processes; iCP, intermediate ciliary process; ppZ, pars plana zonule; VM, vitreous membrane, Z, zonule.

The zonular apparatus in canine eyes demonstrates anatomical and microscopic characteristics that closely mirror those of felines (Figures 8A–C). Similar to the feline configuration, canine eyes possess both major and intermediate CPs (Figure 8B), a feature absent in porcine iridociliary anatomy. As illustrated in Figures 8B,C, zonule fibers originate from both the crests of CPs as well as the valleys between them, subsequently projecting toward the lens capsule. An anterior view of the lens further reveals that zonular fibers consolidate into larger bundles at distances of approximately 0.26–0.5 mm posterior to the anterior edge of the CPs before inserting into the lens capsule (Figure 8A).

When viewed from the posterior aspect of the lens, the aVZ are situated in a distinct anatomical plane, separate from the primary lens zonular apparatus. The spacing of aVZ ranged from 13 to 35 μm, with an average value of approximately 25.77 μm. This spatial arrangement closely parallels that observed in porcine eyes, where the aVZ insert into the lens capsule at similarly positioned sites near the lens equator (Figure 8D). Within this region, clearly defined interfascicular spaces are found between the VM and the CPs.

**FIGURE 9:**
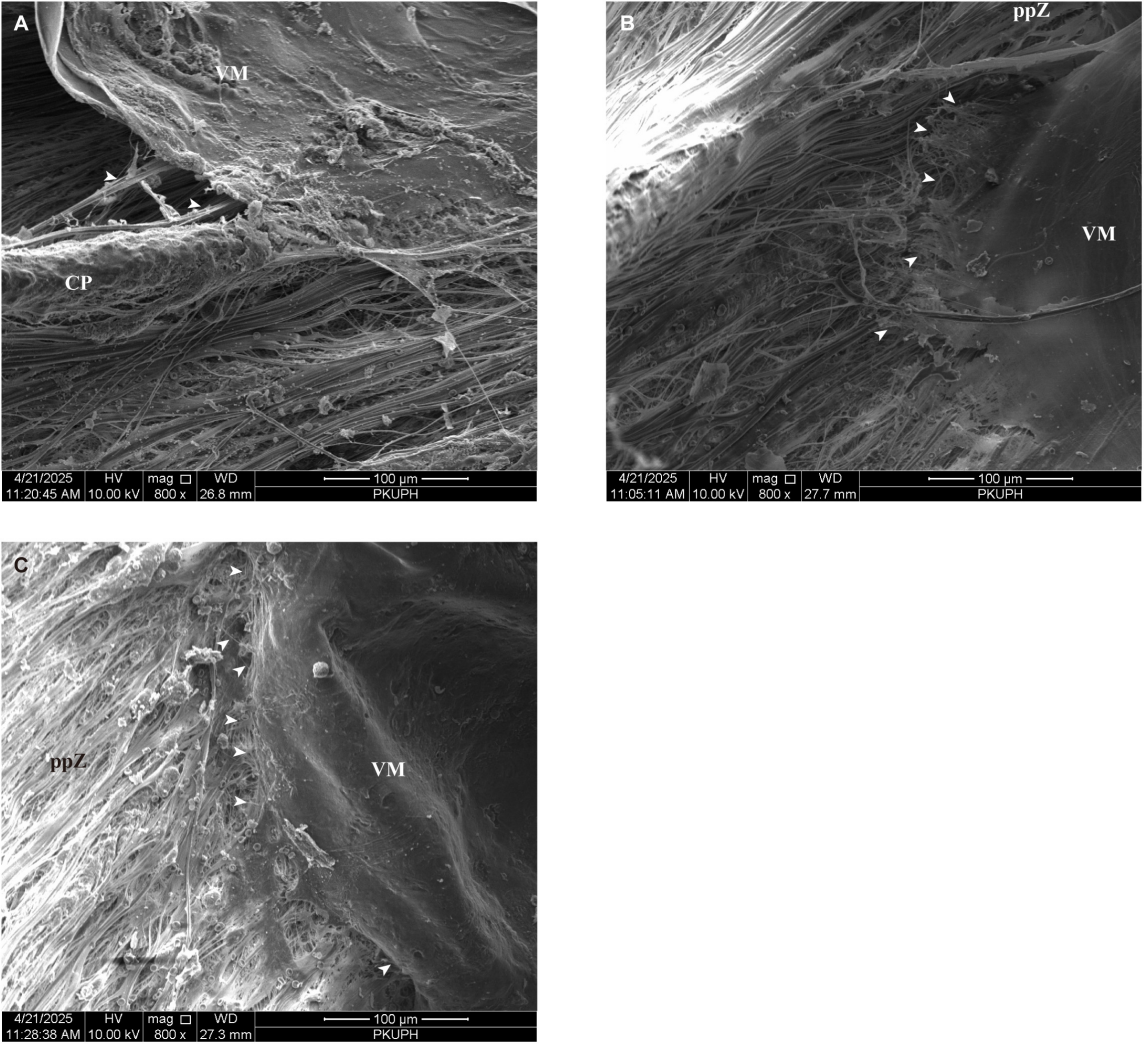
**(A)** Canine eyes display continuous fibrous connections between the vitreous membrane and intermediate vitreous zonules (white arrows). **(B)** Upon dissection of the vitreous membrane from the pars plana to the ora serrata, the posterior vitreous zonules (white arrows) form elaborate interconnections with pars plana zonules. **(C)** Posterior vitreous zonules (white arrows) exhibit an orthogonally interwoven configuration, thereby establishing a complex fibrous matrix. CP, ciliary process; ppZ, pars plana zonule; VM, vitreous membrane.

Further dissection of the VM, extending to the posterior portion of the CPs, reveals the presence of iVZ. Consistent with findings in porcine and feline eyes, the canine iVZ establish fibrous connections with the pars plana zonules. These interconnections exhibit reciprocal weaving, resulting in an intricate reticular network (Figures 8E,F). As observed in other species, this reticular fiber arrangement overlies both the crestal ridges and inter-process valleys of canine CPs (Figures 8E,F). Additionally, canine eyes display continuous fibrous connections between the VM and the iVZ, mirroring the architecture described in porcine and feline specimens (Figure 9A). These iVZ bundles are irregularly spaced around the posterior pars plicata and pars plana at intervals of approximately 32.62μm (ranged from 15.7 to 48.26 μm).

Upon dissection of the VM from the pars plana to the ora serrata, the pVZ form elaborate interconnections with pars plana zonules (Figure 9B). These fibers exhibit an orthogonally interwoven configuration, thereby establishing a complex fibrous matrix (Figure 9C). The porosity of pVZ was measured at 20.95%. As shown in Table 1, the variations in the ocular suspensory zonule system among humans, pigs, cats, and dogs are summarized.

**TABLE 1:** A comparative analysis of zonular apparatus morphology in human, porcine, feline, and canine eyes using SEM and UBM.

	Human	Pig	Cat	Dog
CP Morphology (SEM)	Ellipsoidal cylindrical (9, 10)	Ellipsoidal cylindrical (Figure 4F)	Leaf-like (Figure 6F)	Leaf-like (11) (Figure 8A)
Zonular insertion (SEM)	Valleys of CPs (9, 10)	Valleys and crests of CPs (12) (Figure 3D)	Valleys and crests of CPs (Figures 6C,D)	Valleys and crests of the CPs (11)(Figures 8B,C)
Zonular Fiber packing Density (SEM)	Radially dispersed arrays	Densely clustered (Figure 3D)	Moderate-ly branched network (Figures 6C,D)	Moderately branched network (11) (Figures 8B,C)
AVZ Insertion (SEM)	VM at the posterior LC interface at spatial intervals of approximately 75 μm (1)	VM at the posterior LC interface at intervals of 13.06 μm (Figures 5,C)	Similar to canine eyes at intervals of 27.25 μm (see in Supplementary file)	VM at the posterior lens capsule interface at intervals of 25.77 μm (Figure 8D)
IVZ (SEM)	Regularly spaced arrangement (1)	Compact organization at intervals of 17.62 ± 1.77 μm (Figures 5E)	Irregular, moderately branched structure at intervals of 16.26 ± 2.16 μm (Figures 7E,F)	Irregular, moderately branched structure at intervals of approximately 32.62 μm (Figures 8F, 9A)
PVZ (SEM)	Latticelike structure (1)	Multilayered, interconnected complex with porosity of 20.75% (Figure 5F)	Elaborate interconnections with pars plana zonules with porosity of 30.90% (Figure 7E)	Elaborate interconnections with pars plana zonules with porosity of 20.95% (Figures 9B,C)
Sagittal width of PVZ region width (mm)	3–4 (1)	∼0.56 (Figure 2C)	∼0.4 (Figure 2G)	0.3 (Figure 2K)
VZ visibility (UBM)	Visible (1)	Visible	Visible	Not visible

AVZ, anterior vitreous zonule; CP, ciliary process; LC, lens capsule; IVZ, intermediate vitreous zonule; PVZ, posterior vitreous zonule; SEM, scanning electron microscope; UBM, ultrasound biomicroscopy; VM, vitreous membrane; VZ, vitreous zonule.

## 4 Discussion

Previous research on vitreous zonules has primarily focused on primates, including humans and monkeys (1, 4–6, 9, 13, 14). In comparative morphological studies of non-primate mammal attention has primarily been directed toward the ciliary muscle and microvasculature (15–22), as well as the biomechanical properties of the lens (23–29). Comprehensive studies on the ciliary body morphology and vitreous zonules are scarce. Although earlier research on individual species, such as pigs, cats, and dogs has been reported, comparative studies on the lens suspension systems of pigs, cats, and dogs remain limited, and the presence of vitreous zonules has not been thoroughly elucidated (11, 12, 30–32). To our knowledge, this study presents the first structural characterization of the vitreous zonule system in porcine, feline, and canine eyes, and provides a detailed comparative morphological analysis of the zonular apparatus across these three non-primate mammalian species.

Although Tanimura et al. conducted a comparative analysis of zonular systems in large domestic animals (33) and Yılmaz et al.’s investigated zonular variations in canine cephalic types (32), the structure and organization of the VZ in non-primate mammals have remained unexplored. Early investigations by Coleman employing UBM identified vitreous strands in human subjects (34), while Glasser et al. reported similar findings in primate eyes (35); however, neither study clearly defined the anatomical relationships of these strands to adjacent ocular structures. Furthermore, some researchers identified these strands as the anterior hyaloid membrane (36, 37). It was not until the contribution of Lutjen et al that this structure was formally designated the “vitreous zonule.” They demonstrated that the VZ system orchestrates accommodative biomechanics through dual mechanisms: (1) anchoring anteromedial ciliary muscle displacement via microfibrillar anchoring during contraction, thereby attenuating peripheral retinal strain and reducing the risk of rhegmatogenous detachment; and (2) transducing muscular forces to the lens with phase-locked precision throughout the contraction-relaxation cycles of accommodation. Recent studies have demonstrated that the inability to visualize the VZ using UBM is a key indicator of biomechanical vulnerability in PAC and PACG eyes, directly implicating VZ deficiency in the onset of PACD (4, 5). This study first identifies VZ in non-primate species, providing appropriate animal models for investigating novel pathogenic mechanisms underlying PACG. In addition, previous research has not examined the effects of varying intraocular pressure and the administration of intraocular pressure-lowering medications on the visibility of the vitreous zonule (1, 4–6, 13, 14). Consequently, further studies are warranted to address these gaps.

Non-human primates have been the preferred model in early studies of the zonular system because of their anatomical and genetic similarity to humans (1, 9, 13, 14, 35, 36, 38, 39). However, ethical and financial constraints limit their suitability for large-scale experiments. Rodents (rats and mice), although common in ophthalmic research, have eyes that are too small for interventions on the zonular apparatus. Therefore, for the present study we selected four readily available and cost-effective species: pigs, cats, and dogs.

In this study, microscopy of gross, stained, and UBM-processed specimens provided clear visualization of the primary zonular fiber configuration. This enhanced clarity is attributed to the preservation of zonular structural integrity during examination. In contrast, reliance on single SEM imaging was hindered by artifacts resulting from tension loss during dissection. The application of complementary methodologies, such as employing different techniques on both eyes of the same specimen, yielded the most robust and comprehensive data. Although most studies emphasize differences between feline and canine eyes (33, 40–42), our findings reveal notable similarities in their ciliary bodies and zonular systems. Among the species evaluated, the porcine zonular system differed markedly from the others and showed greater anatomical and microstructural similarity to humans, notably ellipsoidal, cylindrical CPs and a clearly defined aVZ–iVZ–pVZ system. Thus, pigs are a suitable model for investigating the VZ’s role in glaucoma and accommodation. Although the feline CPs differs substantially from that of humans, the presence of a distinct aVZ–iVZ–pVZ system in cats supports their use as a model for VZ-related studies.

Comparative analysis demonstrates that zonular fibers in canine, feline, and porcine eyes exhibit a substantially greater degree of interlacing. In contrast, the apices of CPs in humans remain largely exposed due to the absence of discrete insertion of zonular fibers into the CPs (9, 10, 43–46); such attachments are frequently observed in porcine, feline and canine eyes, particularly on the posterior surface. Furthermore, while pars plana fibers in these species display a meridional orientation similar to that in humans, they extend circumferentially toward the retinal ciliary margin. This is in contrast to human zonular fibers, which converge at the dentate processes of the ora serrata. Collectively, these anatomical differences suggest that, canine, feline, and porcine eyes possess a reduced capacity for accommodative function compared to humans (20), but may benefit from enhanced mechanical buffering and increased structural stability of the lens.

Due to the anatomical and biomechanical similarities to human eyes, especially in lens properties, porcine eyes are frequently utilized in presbyopia research (26–29). Conversely, feline eyes are naturally myopic, potentially masking presbyopic symptoms and making them less suitable for presbyopia studies (47). The refractive states in dogs vary significantly among breeds; Dachshunds, Collies, Miniature Schnauzers, and Toy Poodles are typically myopic, whereas Australian Shepherds, Alaskan Malamutes, and Bouvier des Flandres tend to be hyperopic (48). Studies have shown that the degree of myopia in dogs increases with age, suggesting that canine eyes may also be unsuitable for presbyopia research (48).

In primates, the anterior VM and the posterior zonular fibers of the anterior zonular fork (attached to the posterior lens capsule) are anatomically separated, an arrangement that likely facilitates unrestricted lens movement (1). Our SEM studies revealed a similar detachment between the anterior VZs and the zonular fibers in porcine and canine eyes. In addition, the VM adjacent to the lens in feline specimens was readily displaceable. These findings parallel the iVZ configuration observed in primates, indicating a conserved anatomical feature among these species that enables unimpeded lens dynamics.

The iVZ could contribute to preserving the anatomical configuration of the VM while regulating anteromedial ciliary muscle dynamics during accommodation (1, 6). In primates, anterior iVZ segments display regular spacing corresponding to the dimensions of the CPs as they diverge anteriorly to anchor within the interciliary valleys. Notably, our observations in animal models (porcine, feline, and canine eyes) reveal a distinct arrangement: iVZ fibers extend anteriorly to both the CPs (ciliary crests) and the interciliary valleys, where they attach to the hyaloid membrane. Such an anatomical configuration may represent a relatively diminished role in accommodative function compared to primates, while providing enhanced structural stabilization and mechanical damping.

The pVZ reduces mechanical strain on the peripheral retina during accommodation (1). The enhanced dimensional characteristics (length and thickness) of the human vitreoretinal interface, relative to simian, porcine, feline and canine counterparts, likely represent an evolutionary adaptation providing greater retinal protection throughout the extended human lifespan (1).

The iVZ fibers are readily visualized using high-resolution UBM (4). Consistent with this, UBM imaging of *ex vivo* porcine and feline eyes clearly revealed the presence of the iVZ. However, the iVZ was not clearly discernible in UBM examinations of canine eyes, which may result from the limited sample sizes. Alternatively, anatomical differences may play a role: previous human studies have shown that narrower iridocorneal angles are associated with less VZ (4, 5). Recent comparative studies indicate that canine eyes possess narrower iridocorneal angles than feline eyes (41, 42), potentially making VZ identification more challenging in the canine model.

Study limitations included a restricted cohort size for canines and the absence of quantitative assessment for zonular parameters across species. Nonetheless, comparative analyses permitted evidence-based selection of appropriate animal models. To fully elucidate the vitreous zonules biomechanics in pigs and cats, future studies should integrate data from primate eye models—specifically measurements of dynamic stretch responses and energy dissipation efficiency. Such comparative analyses will assess how faithfully these species replicate human accommodation, presbyopia, and glaucoma mechanisms and will support translational applications.

## 5 Conclusion

Using UBM, H&E staining, Masson’s trichrome staining, and SEM, we analyzed the zonular apparatus in porcine, feline, and canine eyes. Among the species examined, porcine eyes exhibited the closest morphological resemblance to human eyes. They featured ellipsoidal cylindrical ciliary processes, a distinct anterior–intermediate–posterior VZ system, and a posterior vitreous zonule region width of approximately 0.56 mm, which is substantially more consistent with human measurements than those found in feline or canine eyes. Although the feline CPs differs substantially from that of humans, the presence of a distinct aVZ–iVZ–pVZ system in cats supports their use as a model for VZ-related studies. Unique zonular fiber origins and the ubiquitous VZ support the use of pigs or cats in studies of accommodation and glaucoma pathogenesis.

## Data Availability

The original contributions presented in the study are included in the article/Supplementary material, further inquiries can be directed to the corresponding author.

## References

[1] Lütjen-Drecoll E Kaufman PL Wasielewski R Ting-Li L Croft MA. Morphology and accommodative function of the vitreous zonule in human and monkey eyes. *Invest Ophthalmol Vis Sci*. (2010) 51:1554–64. 10.1167/iovs.09-4008 19815737 PMC2829378

[2] Pan Y Liu Z Zhang H. Research progress of lens zonules. *Adv Ophthalmol Pract Res*. (2023) 3:80–5. 10.1016/j.aopr.2023.02.002 37846380 PMC10577871

[3] Huang J Huang C. Zonulopathy and its relation to primary angle closure disease: a review. *J Glaucoma*. (2024) 33:931–9. 10.1097/IJG.0000000000002385 38573908

[4] Lv K Liang Z Yang K Chen X Ma Y Wu H. Novel discoveries of the relationship between the vitreous zonule and the anterior segment characteristics in eyes with primary angle-closure disease. *Invest Ophthalmol Vis Sci*. (2022) 63:16. 10.1167/iovs.63.13.16 36520454 PMC9769030

[5] Shon K Sung KR Kwon J Hye Jo Y. Vitreous zonule and its relation to anterior chamber angle characteristics in primary angle closure. *J Glaucoma*. (2019) 28:1048–53. 10.1097/IJG.0000000000001387 31633619

[6] Kaufman PL Lütjen Drecoll E Croft MA. Presbyopia and Glaucoma: two diseases, one pathophysiology? The 2017 friedenwald lecture. *Invest Ophthalmol Vis Sci*. (2019) 60:1801–12. 10.1167/iovs.19-26899 31038661 PMC6540935

[7] Wu D Liu Y Zhang X Zhang R Wang S Lu H Efficacy and safety of stem cells in the treatment of glaucoma: systematic review and meta-analysis based on animal experiments. *Front Pharmacol*. (2025) 16:1587440. 10.3389/fphar.2025.1587440 40667506 PMC12259681

[8] Becker S L’Ecuyer Z Jones BW Zouache MA McDonnell FS Vinberg F. Modeling complex age-related eye disease. *Prog Retin Eye Res*. (2024) 100:101247. 10.1016/j.preteyeres.2024.101247 38365085 PMC11268458

[9] Rohen JW. Scanning electron microscopic studies of the zonular apparatus in human and monkey eyes. *Invest Ophthalmol Vis Sci.* (1979) 18:133–44. 104933

[10] Canals M Costa-Vila J Potau JM Merindano MD Ruano D. Scanning electron microscopy of the human zonule of the lens (*Zonula ciliaris*). *Acta Anat*. (1996) 157:309–14. 10.1159/000147893 9259880

[11] Curtis R. The suspensory apparatus of the canine lens. *J Anat* (1983) 136(Pt 1):69–83. 6833122 PMC1171930

[12] Bãlãşoiu M Bãlãşoiu AT Mãnescu R Avramescu C Ionete O. *Pseudomonas aeruginosa* resistance phenotypes and phenotypic highlighting methods. *Curr Health Sci J*. (2014) 40:85–92. 10.12865/CHSJ.40.02.01 25729587 PMC4340447

[13] Croft MA McDonald JP Katz A Lin TL Lütjen-Drecoll E Kaufman PL. Extralenticular and lenticular aspects of accommodation and presbyopia in human versus monkey eyes. *Invest Ophthalmol Vis Sci*. (2013) 54:5035–48. 10.1167/iovs.12-10846 23745002 PMC3726241

[14] Croft MA Nork TM McDonald JP Katz A Lütjen-Drecoll E Kaufman PL. Accommodative movements of the vitreous membrane, choroid, and sclera in young and presbyopic human and nonhuman primate eyes. *Invest Ophthalmol Vis Sci*. (2013) 54:5049–58. 10.1167/iovs.12-10847 23745005 PMC3726242

[15] Gillum W. Mechanisms of accommodation in vertebrates. *Ophthalmic Semin.* (1976) 1:253–86. 828713

[16] Morrison JC DeFrank MP Van Buskirk EM. Comparative microvascular anatomy of mammalian ciliary processes. *Invest Ophthalmol Vis Sci.* (1987) 28:1325–40. 3610551

[17] McDonald TF Cheeks L Slagle T Green K. Marijuana-derived material-induced changes in monkey ciliary processes differ from those in rabbit ciliary processes. *Curr Eye Res*. (1991) 10:305–12. 10.3109/02713689108996336 2070639

[18] Funk RH. The vessel architecture of the pars plana in the cynomolgus monkey, rat and rabbit eye. A scanning electron microscopic study of plastic corrosion casts. *Ophthalmic Res*. (1993) 25:337–48. 10.1159/000267335 8309672

[19] Natiello M Lewis P Samuelson D. Comparative anatomy of the ciliary body of the West Indian manatee (*Trichechus manatus*) and selected species. *Vet Ophthalmol*. (2005) 8:375–85. 10.1111/j.1463-5224.2005.00408.x 16359360

[20] Ott M. Visual accommodation in vertebrates: mechanisms, physiological response and stimuli. *J Comp Physiol A Neuroethol Sens Neural Behav Physiol*. (2006) 192:97–111. 10.1007/s00359-005-0049-6 16172892

[21] Sedacca K Samuelson D Lewis P. Examination of the anterior uveoscleral pathway in domestic species. *Vet Ophthalmol*. (2012) 15(Suppl 1):1–7. 10.1111/j.1463-5224.2011.00914.x 22051120

[22] Rodriguez-Ramos Fernandez J Dubielzig RR. Ocular comparative anatomy of the family Rodentia. *Vet Ophthalmol*. (2013) 16(Suppl 1):94–9. 10.1111/vop.12070 23734597

[23] Willekens B Vrensen G. Lens fiber organization in four avian species: a scanning electron microscopic study. *Tissue Cell*. (1985) 17:359–77. 10.1016/0040-8166(85)90055-2 4012767

[24] Schachar RA Pierscionek BK Abolmaali A Le T. The relationship between accommodative amplitude and the ratio of central lens thickness to its equatorial diameter in vertebrate eyes. *Br J Ophthalmol*. (2007) 91:812–7. 10.1136/bjo.2006.107524 17050574 PMC1955594

[25] Sharma PK Busscher HJ Terwee T Koopmans SA van Kooten TGA. comparative study on the viscoelastic properties of human and animal lenses. *Exp Eye Res*. (2011) 93:681–8. 10.1016/j.exer.2011.08.009 21910988

[26] Kammel R Ackermann R Mai T Damm C Nolte S. Pig lenses in a lens stretcher: implications for presbyopia treatment. *Optom Vis Sci*. (2012) 89:908–15. 10.1097/OPX.0b013e318255da87 22561204

[27] Reilly MA Martius P Kumar S Burd HJ Stachs O. The mechanical response of the porcine lens to a spinning test. *Z Med Phys*. (2016) 26:127–35. 10.1016/j.zemedi.2015.12.009 26777319

[28] Wang K Qiu Z Xie Y Cai S Zhao Y Pierscionek BK Design of an automatically controlled multi-axis stretching device for mechanical evaluations of the anterior eye segment. *Bioengineering*. (2023) 10:142. 10.3390/bioengineering10020142 36829636 PMC9952546

[29] Crews M Rich W Reilly MA. Influence of zonular tension on molecular transport in the porcine ocular lens. *Front Ophthalmol*. (2024) 4:1508779. 10.3389/fopht.2024.1508779 39687503 PMC11646982

[30] Hoyer HE. Scanning electron-microscopic study of lens fibers of the pig. *Cell Tissue Res*. (1982) 224:225–32. 10.1007/BF00217281 7094010

[31] Halenda RM Nasisse MP Dykstra MJ. Effect of alpha-chymotrypsin on breaking strength and ultrastructural morphology of canine ciliary zonules. *Am J Vet Res.* (1998) 59:335–9. 9522954

[32] Yılmaz C Kabak M Selviler Sizer S. Comparative macroanatomical and scanning electron microscopy study of the eyeball in brachycephalic and mesocephalic dog breeds. *Microsc Res Tech*. (2024) 87:2408–17. 10.1002/jemt.24624 38822703

[33] Tanimura I. [Comparative morphology of the bulbus oculi of the domestic animals revealed by scanning electron microscopy (author’s transl)]. *Nihon Juigaku Zasshi*. (1977) 39:643–56. 10.1292/jvms1939.39.643 599762

[34] Coleman DJ Silverman RH Lizzi FL Lloyd H Rondeau MJ. *Ultrasonography of the Eye and Orbit.* 2nd ed. Philadelphia: Lippincott Williams & Wilkins (2006).

[35] Glasser A Croft MA Brumback L Kaufman PL. Ultrasound biomicroscopy of the aging rhesus monkey ciliary region. *Optom Vis Sci*. (2001) 78:417–24. 10.1097/00006324-200106000-00014 11444631

[36] Bernal A Parel JM Manns F. Evidence for posterior zonular fiber attachment on the anterior hyaloid membrane. *Invest Ophthalmol Vis Sci*. (2006) 47:4708–13. 10.1167/iovs.06-0441 17065477

[37] Byrne SFGR. *Ultrasound of the Eye and Orbit.* 2nd ed. St. Louis: Mosby, Inc (2002).

[38] Glasser A Kaufman PL. The mechanism of accommodation in primates. *Ophthalmology*. (1999) 106:863–72. 10.1016/S0161-6420(99)00502-3 10328382

[39] Croft MA Heatley G McDonald JP Katz A Kaufman PL. Accommodative movements of the lens/capsule and the strand that extends between the posterior vitreous zonule insertion zone & the lens equator, in relation to the vitreous face and aging. *Ophthalmic Physiol Opt*. (2016) 36:21–32. 10.1111/opo.12256 26769326 PMC4755275

[40] Coile DC O’Keefe LP. Schematic eyes for domestic animals. *Ophthalmic Physiol Opt*. (1988) 8:215–20. 10.1111/j.1475-1313.1988.tb01040.x 3211562

[41] Kim D Jung JS Hwang J Park J Kwon M Yong J Comparative analysis of iridocorneal angle in cats and dogs using ultrasound biomicroscopy: implications for glaucoma prevalence. *BMC Vet Res*. (2025) 21:181. 10.1186/s12917-025-04648-5 40102853 PMC11921709

[42] Kim D Kwon H Hwang J Jung JS Park KM. An in-depth review on utilizing ultrasound biomicroscopy for assessing the iridocorneal angle and ciliary body in canines. *Front Vet Sci*. (2025) 12:1501405. 10.3389/fvets.2025.1501405 40078210 PMC11897051

[43] Mcculloch C. The zonule of Zinn: its origin, course, and insertion, and its relation to neighboring structures. *Trans Am Ophthalmol Soc.* (1954) 52:525–85. 13274438 PMC1312608

[44] Vail D. The zonule of Zinn and ligament of Wieger; their importance in the mechanics of the intracapsular extraction of cataract. *Trans Ophthalmol Soc U K* (1957) 77:441–99. 13530128

[45] Rohen JW Rentsch FJ. [Architecture and function of the human zonula ciliaris. Morphological principles for a new theory of accommodation]. *Albrecht Von Graefes Arch Klin Exp Ophthalmol*. (1969) 178:1–19. 10.1007/BF00428041 5306789

[46] Farnsworth PN Burke P. Three-dimensional architecture of the suspensory apparatus of the lens of the Rhesus monkey. *Exp Eye Res*. (1977) 25:563–76. 10.1016/0014-4835(77)90135-x 412688

[47] Konrade KA Hoffman AR Ramey KL Goldenberg RB Lehenbauer TW. Refractive states of eyes and associations between ametropia and age, breed, and axial globe length in domestic cats. *Am J Vet Res*. (2012) 73:279–84. 10.2460/ajvr.73.2.279 22280390

[48] Kubai MA Bentley E Miller PE Mutti DO Murphy CJ. Refractive states of eyes and association between ametropia and breed in dogs. *Am J Vet Res*. (2008) 69:946–51. 10.2460/ajvr.69.7.946 18593249

