# Supplementary figures and images for: Comparative morphological analysis of the zonular apparatus in porcine, feline, and canine species
Source: Front Med (Lausanne). 2026 Jan 14;12:1717670. doi: 10.3389/fmed.2025.1717670 (PMC12847380; doi:10.3389/fmed.2025.1717670)

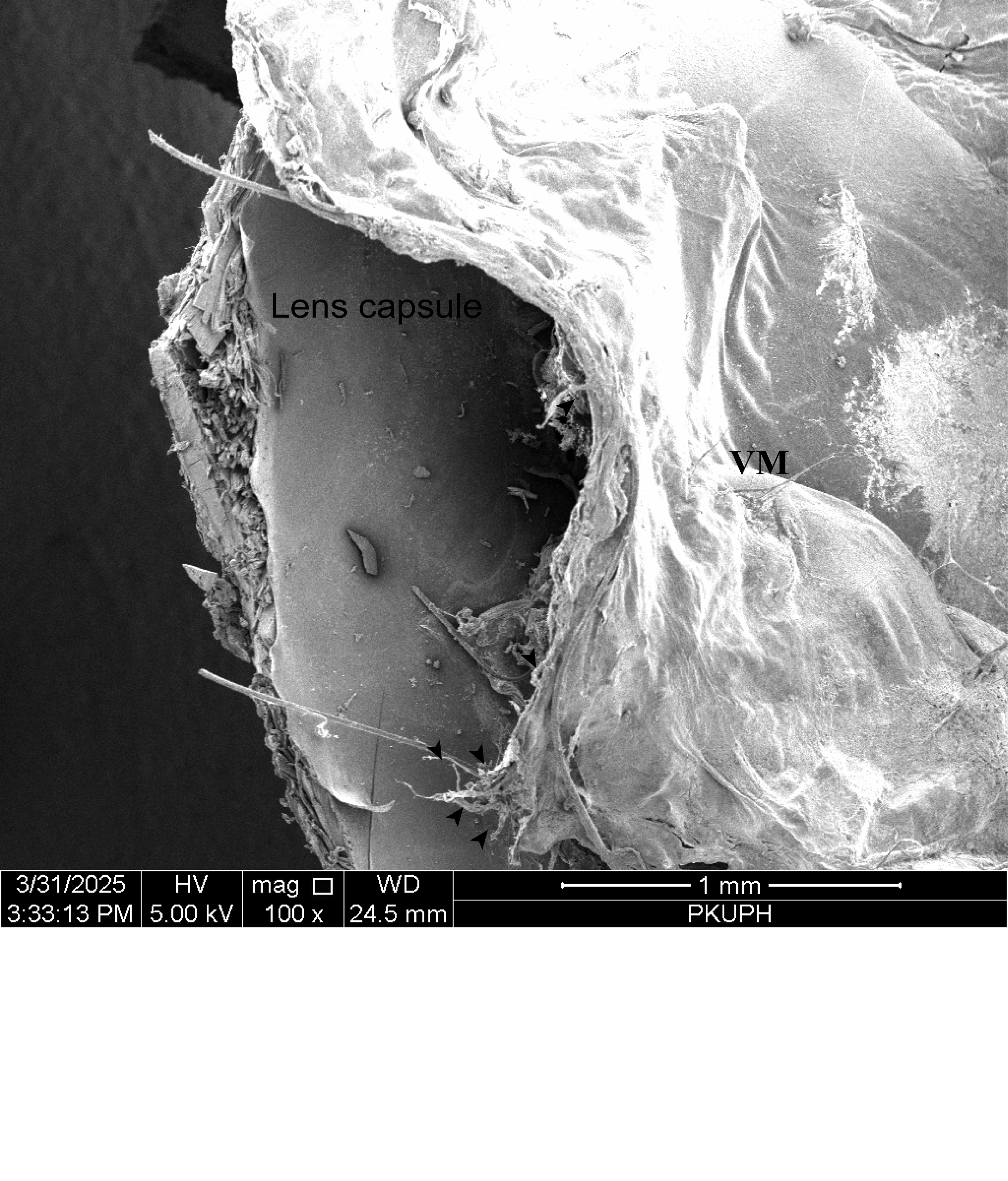

Supplement: Supplementary file 1 [file Image_1.tif]
